# A Case of Idiopathic Chest Pain Following Watchman Device Placement

**DOI:** 10.1002/ccr3.70679

**Published:** 2025-07-27

**Authors:** Salman Pervaiz, Abeera Azam, Masab Ali, Muhammad Husnain Ahmad, Preetham Muskula, Tyrone Galbreath, Muhammad Hassan

**Affiliations:** ^1^ Davis Heart and Lung Institute Columbus Ohio USA; ^2^ The Ohio State University Wexner Medical Center Columbus Ohio USA; ^3^ Department of Internal Medicine UT Health East Texas Tyler Texas USA; ^4^ Department of Medicine Punjab Medical College Punjab Pakistan; ^5^ Department of Medicine Tentishev Satkynbai Memorial Asian Medical Institute Kant Kyrgyzstan; ^6^ Department of Cardiology UT Health East Texas Tyler Texas USA; ^7^ Department of Cardiothoracic Surgery UT Health East Texas Tyler Texas USA

**Keywords:** atrial appendage, chest pain, left atrial appendage closure, watchman

## Abstract

Chest pain following left atrial appendage closure (LAAC) with the Watchman device is rare but can occur due to complications such as pericarditis or myocardial infarction. We present the case of a 59‐year‐old male with atrial fibrillation who developed sharp, left‐sided chest pain exacerbated by inspiration after undergoing Watchman device placement. Despite extensive diagnostic evaluations, including electrocardiograms (ECGs), echocardiograms, and cardiac catheterization, the exact cause of the pain remained unclear, with no signs of acute coronary syndrome or pericardial effusion. The patient's symptoms persisted despite treatment with colchicine and anti‐inflammatory agents including steroids. Due to the severe and persistent nature of the pain, the patient opted for surgical removal of the Watchman device. This procedure led to a significant resolution of his symptoms. This case underscores the need to consider device removal for persistent, idiopathic chest pain after LAA occlusion, especially when other causes are excluded and conservative management fails.

**Summary**

- Idiopathic chest pain refractory to medical treatment is a rare complication of Watchman device placement in the absence of any effusion. Surgical removal of the LAAC device should be considered in patients with sharp post‐procedural chest pain causing functional impairment.

## 1 Introduction

LAAC has emerged as an effective non‐pharmacological therapy in patients with non‐valvular atrial fibrillation for stroke prophylaxis in recent years [1, 2]. Traditionally, warfarin and other oral anticoagulants have been used for prophylaxis against thromboembolic events in patients with atrial fibrillation, but warfarin predisposes one to an increased bleeding risk. Non‐vitamin K oral anticoagulants (NOACs), especially apixaban, have a favorable risk profile compared to warfarin. Still, there remains an elevated risk of intracranial bleeding and gastrointestinal bleeding with their use [3]. Numerous trials, such as the PREVAIL trial, have established the efficacy and non‐inferiority of the Watchman device [4]. There remains a risk of peri‐procedural and late complications such as myocardial infarction, peri‐device leakage, device embolization, pericardial effusion, and cardiac tamponade with LAA occlusion devices [2, 3]. We report a rare case of sharp pleuritic chest pain after LAA occlusion with a Watchman device, refractory to medical management, which ultimately necessitated the device's removal.

## 2 Case Presentation

We present the case of a 59‐year‐old male with a cardiovascular history of paroxysmal atrial fibrillation (AF) status post ablation, followed by stroke prevention with oral apixaban. The patient reported no recurrent AF symptoms after the ablation. However, 4 months later, thromboprophylaxis with apixaban was complicated by multiple episodes of gastrointestinal (GI) bleeding. This led to the proposal of LAA occlusion with a Watchman device. He subsequently underwent device placement at an outside facility. Immediately after the procedure, he developed left‐sided chest pain, described as sharp, which worsened with inspiration. The patient's medical history is significant for paroxysmal atrial fibrillation, non‐ischemic cardiomyopathy, heart failure with reduced ejection fraction (EF) of ~35%–40% recovered to 50%–55%, hypertension, and hyperlipidemia.

## 3 Differential Diagnosis and Investigations

The differential diagnosis included pericardial effusion, pericarditis, pleurisy, acute coronary syndrome (ACS), and vasospastic angina. ECGs from subsequent ER visits ruled out ACS. Transesophageal echocardiogram (TTE) confirmed the Watchman device was correctly positioned in the left atrial appendage (LAA) with no pericardial or pleural effusion. Chest radiograph and CT scans were unremarkable. Cardiac CT with contrast confirmed proper device placement in the LAA and showed no contrast passage beyond the Watchman, indicating no leakage. Cardiac catheterization revealed borderline right coronary artery (RCA) stenosis. Laboratory tests showed normal troponin, electrolytes, complete blood count, and inflammatory markers.

## 4 Management

**FIGURE 1:**
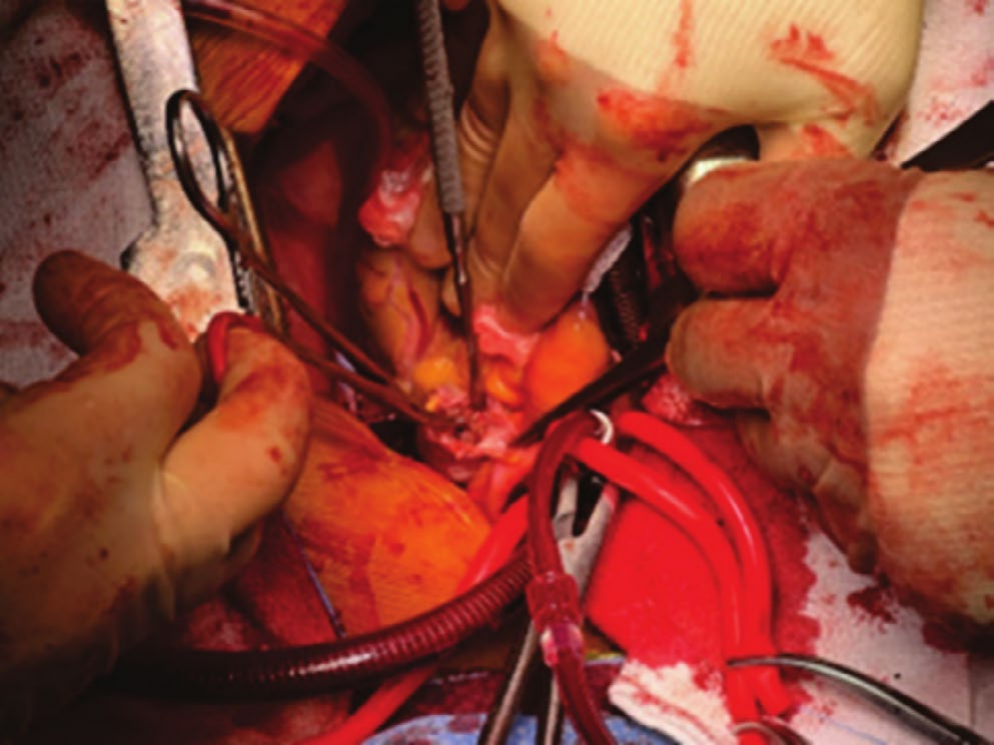
Surgical removal of the Watchman device. Ridge of LAA was opened and Freer's elevator was used to circumferentially lyse adhesions.

**FIGURE 2:**
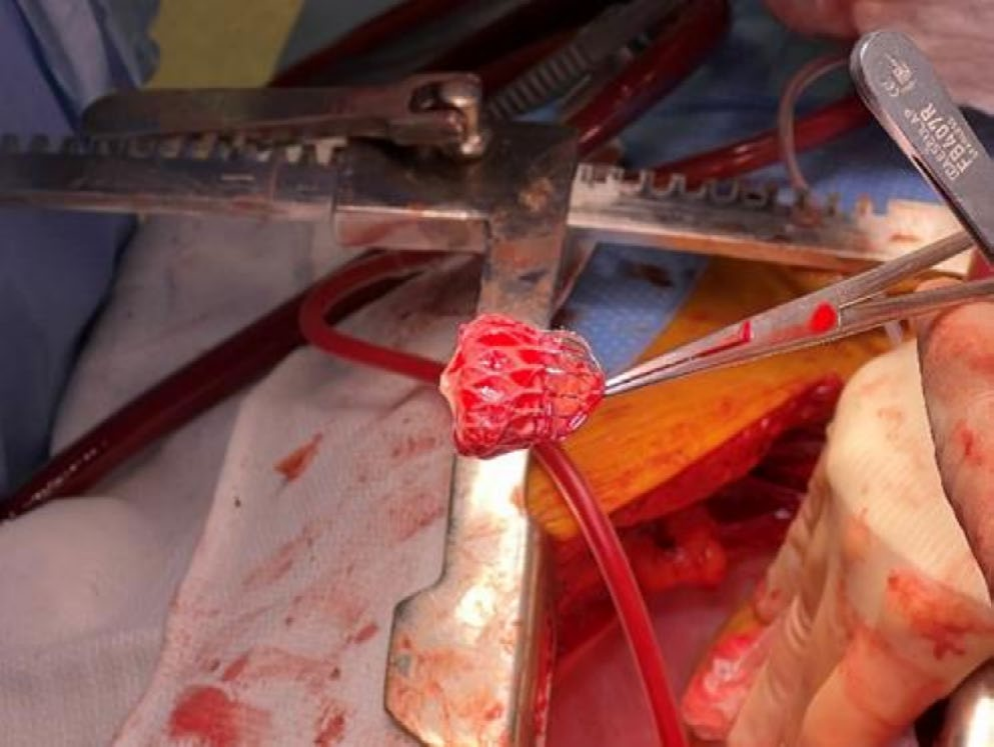
Partially endothelialized Watchman device. Surgical retrieval of the partially endothelialized Watchman device without any evidence of peridevice leak on TTE.

The sharp chest pain continued for several weeks, warranting multiple ER visits. Each time, he was ruled out for acute coronary syndrome. His primary electrophysiologist then saw him, and an echocardiogram revealed normal EF and negative for pericardial effusion. During this period, the patient remained afebrile. Due to suspicion of pericarditis, he was started on colchicine, ibuprofen, and later prednisone taper. Despite this, the pain persisted. On his third ER visit for the persistent unresolved chest pain, he was eventually transferred to our facility for a higher level of care. He was taken for urgent cardiac catheterization and found to have single‐vessel right RCA with borderline stenosis. A multidisciplinary team involving an electrophysiologist and cardiothoracic surgeon visited with the patient, and after extensive discussion, the patient ultimately opted for the removal of the device via sternotomy. Two months later, the patient underwent elective removal of the Watchman device followed by a Maze procedure (Figure 1). No evidence of inflammation was seen surrounding the appendage (Figure 2). The native tissue was gently dissected from the device and extracted from the left atrial appendage. Subsequently, a modified maze procedure was performed.

## 5 Outcome and Follow‐Up

After successful closure, the patient was transferred to the critical care unit. In the following days, he denied chest pain, palpitations, or nausea and participated well in physical therapy. The patient was discharged on the fourth postoperative day in a stable, pain‐free condition with plans to start anticoagulation when deemed clinically stable. The patient stated that he is doing well on his 2‐week post‐op call. The patient had a nurse visit scheduled next week for an incision site examination. He reported a reaction to the Band‐Aid that the family used over the incision but no chest pain.

## 6 Discussion

Chest pain secondary to pericarditis, pericardial effusion, or myocardial infarction is a rare but well‐known complication of left atrial appendage occlusion procedures. We report a case presenting with sharp, left‐sided chest pain, aggravated by inspiration, immediately after the placement of the watchman device. The precise cause of the chest pain in this case remains idiopathic, given the lack of definitive findings on all performed diagnostic tests. To our knowledge, this is the first case of such nature, as the pain did not respond to colchicine, nonsteroidal anti‐inflammatory drugs (NSAIDs), and steroids. The patient ultimately opted for the surgical removal of the transplanted device owing to the severe functional impairment caused by the pain.

Pericarditis secondary to micro‐perforations of the pericardium and delayed pericardial effusion has been linked to the placement of the watchman device. However, the inflammation responded to a short course of colchicine and steroids [5]. A single‐center prospective analysis of 369 patients, who underwent LAAC, revealed that delayed effusions are uncommon and mostly related to device micro‐perforation. Although no statistically significant association was found between the risk factors and the outcome, GI bleeding was the most common indication for undergoing LAAC [6]. In this case, the patient had a history of multiple GI bleeding episodes, but no pericardial or pleural effusion was found, and the pain did not respond to colchicine and anti‐inflammatory agents, including steroids. Earlier, there was a reported case of recurrent left pleural effusion in the setting of pericardial effusion and pericarditis. The patient complained of chest pain while taking colchicine and NSAIDs but was eventually pain‐free after 8 weeks [7].

Alternatively, the pain in this case could be attributed to vasospastic angina. Vasospastic angina has been linked to coronary artery vasospasm, endothelial dysfunction, and reduced nitric oxide bioavailability [8]. Our patient's risk factors, such as hypertension and atherosclerosis, increase the likelihood of developing endothelial dysfunction. A rare case of inferior wall ST‐elevation with reciprocal ST‐segment depression in the lateral wall secondary to coronary artery vasospasm during a combined left atrial appendage closure plus radiofrequency catheter ablation has been reported [9]. Our patient's ECG findings and normal cardiac troponin T (cTNT) during the subsequent ER visits ruled out any acute coronary event.

Borderline coronary lesions are defined as those having 40%–70% of stenosis. Borderline coronary stenosis, as seen in our patient, could lead to the development of ACS but would be evident in ECG findings [10]. Despite its utility in assessing cardiac inflammation and function, cardiac magnetic resonance imaging (MRI) was not utilized in this case. While MRI offers detailed imaging capabilities, it was not accessible for routine evaluations in this instance.

Surgical removal of the Watchman device is usually indicated in cases of device embolization, but should be considered in patients with idiopathic, refractory chest pain causing functional impairment. Modified Maze procedure was performed on this patient after the removal of the Watchman device. Developed by Dr. James Cox, the traditional Maze procedure involves creating surgical incisions to disrupt abnormal electrical pathways causing AF. The modified Maze procedure simplifies this with cryoablation or radiofrequency ablation, which reduces complexity and recovery time. This approach is notable for its high success rate in achieving long‐term rhythm control and reducing AF recurrence [11].

As far as possible, the hypothesis into chest pain in this patient is considered. We can hypothesize that phrenic nerve irritation might cause referred pain to the chest. As we know, the left atrial appendage lies near the phrenic nerve [12], and the catheter or device delivery sheath used to position the Watchman device may come into contact with the phrenic nerve, causing irritation and subsequent chest pain. Similarly, the heart and esophagus are closely related [13] and the Watchman device placement in the left atrial appendage can cause esophageal irritation leading to chest pain. We did not perform provocative maneuvers to see if this is the underlying cause of chest pain. Therefore, we can only hypothesize that this may be the underlying cause of ongoing symptoms, as it causes chest pain [14]. This represents a limitation of the case report.

Watchman device removal is most commonly performed in cases of device embolization [15] or migration [16]. In this case, the device was removed due to functional impairment due to chest pain. Persistent chest pain, which might be due to phrenic nerve irritation caused by the device, and symptoms unresponsive to non‐surgical interventions led to the decision to remove the device after considerable consideration.

This case highlights the importance of considering device removal in patients experiencing severe, persistent chest pain after LAAC when other causes have been ruled out, and pain management remains ineffective.

## 7 Conclusion

We report a rare case of sharp chest pain after placement of the Watchman device, relieved after the surgical removal of the device. More such cases should be reported to determine any commonality in the underlying risk factors of such patients. This will lead to a better understanding of the underlying pathophysiology of this rare occurrence and serve as a guide for different management options.

## Author Contributions

**Salman Pervaiz:** conceptualization, formal analysis, project administration, resources, supervision, writing – original draft. **Abeera Azam:** investigation, project administration, resources, supervision, writing – original draft. **Masab Ali:** conceptualization, formal analysis, project administration, supervision, validation, visualization, writing – original draft, writing – review and editing. **Preetham Muskula:** resources, software, visualization, writing – original draft. **Tyrone Galbreath:** data curation, investigation, project administration, writing – original draft. **Muhammad Husnain Ahmad:** validation, visualization. **Muhammad Hassan:** visualization, writing – original draft, writing – review and editing.

## Consent

Written informed consent was obtained from the patient to publish this report in accordance with the journal's patient consent policy.

## Conflicts of Interest

The authors declare no conflicts of interest.

## Data Availability

Data and materials available upon request from corresponding author.
